# Laser Melting Deposition Additive Manufacturing of Ti6Al4V Biomedical Alloy: Mesoscopic In-Situ Flow Field Mapping via Computational Fluid Dynamics and Analytical Modelling with Empirical Testing

**DOI:** 10.3390/ma14247749

**Published:** 2021-12-15

**Authors:** Muhammad Arif Mahmood, Asif Ur Rehman, Fatih Pitir, Metin Uymaz Salamci, Ion N. Mihailescu

**Affiliations:** 1 National Institute for Laser, Plasma and Radiation Physics (INFLPR), Magurele, 077125 Ilfov, Romania; ion.mihailescu@inflpr.ro; 2 ERMAKSAN, Bursa 16065, Turkey; faith.pitir@ermaksan.com.tr; 3 Department of Mechanical Engineering, Gazi University, Ankara 06570, Turkey; msalamci@gazi.edu.tr; 4 Additive Manufacturing Technologies Research And Application Center-EKTAM, Gazi University, Ankara 06560, Turkey; 5 Manufacturing Technologies Center of Excellence-URTEMM A.S., Ankara 06980, Turkey

**Keywords:** 3D printing, laser melting deposition, computational fluid dynamics model, analytical model, melt flow, marangoni force, recoil pressure

## Abstract

Laser melting deposition (LMD) has recently gained attention from the industrial sectors due to producing near-net-shape parts and repairing worn-out components. However, LMD remained unexplored concerning the melt pool dynamics and fluid flow analysis. In this study, computational fluid dynamics (CFD) and analytical models have been developed. The concepts of the volume of fluid and discrete element modeling were used for computational fluid dynamics (CFD) simulations. Furthermore, a simplified mathematical model was devised for single-layer deposition with a laser beam attenuation ratio inherent to the LMD process. Both models were validated with the experimental results of Ti6Al4V alloy single track depositions on Ti6Al4V substrate. A close correlation has been found between experiments and modelling with a few deviations. In addition, a mechanism for tracking the melt flow and involved forces was devised. It was simulated that the LMD involves conduction-mode melt flow only due to the coaxial addition of powder particles. In front of the laser beam, the melt pool showed a clockwise vortex, while at the back of the laser spot location, it adopted an anti-clockwise vortex. During printing, a few partially melted particles tried to enter into the molten pool, causing splashing within the melt material. The melting regime, mushy area (solid + liquid mixture) and solidified region were determined after layer deposition. This research gives an in-depth insight into the melt flow dynamics in the context of LMD printing.

## 1. Introduction

Additive manufacturing (AM) provides customized designs, reduces preparation time, and produces complicated shapes. Many advanced technological applications [1], aerospace [2], biomedicine [3,4] and architecture [5,6] have grabbed interest in it. Laser additive manufacturing (LAM) is a subtype of additive manufacturing (AM) that fuses the powder particles with a laser beam to generate high-quality metallic parts [7]. LAM has the quickest annual growth of all AM methods and is used in various industries, including automobile, space, healthcare and energy [8,9,10,11,12,13]. Laser metal deposition (LMD) is a sub-branch of AM with various applications, including surface treatment and coatings [14], the production of functionally graded materials [15] and restoration of broken parts [16]. One additional benefit of employing the LMD methodology is its short production period and reduced waste of materials, opposite to conventional manufacturing techniques [17]. Furthermore, Ya et al. [18], Froend et al. [19], Jhavar et al. [20] and Bailey et al. [21] demonstrated that worn-out metallic components restored using LMD presented outstanding structural characteristics and longer in-service time than traditional processes. In the LMD process, utilizing the carrier gases, metal microparticles having a diameter of approximately 30–100 µm are deposited onto the substrate. Simultaneously, a laser beam was applied to melt the metallic powder particles, thus yielding the continuous layers. Zhu et al. [22] and Wang et al. [23] reported the volume and shape of the formed tracks governed the result of a sample’s production. In the LMD method, various input variables exist, including beam power, scan speed, and powder flow rate, which can significantly affect the process, and consequently, the integrity of the finished objects [24,25]. Process parameters optimization can be time-consuming and expensive due to the hit-and-trial technique usually implemented to determine the operating conditions [26]. Post-characterization techniques such as scanning electron microscopy, electron backscatter diffraction (EBSD) and x-ray computed tomography (XCT) are not capable of providing information about heat and fluid flow [27].

An effective tool for in-situ monitoring the LMD process is via “operando” monitoring, which is usually achieved using photodetector or thermal infrared imaging. However, even these techniques will not expose the internal liquid fluid dynamics and temperature distribution throughout the process. The thermal-imaging fails to collect the fluid metal’s surface heat accurately due to emissivity alternation drastically during the phase transformation [19]. A more beneficial way to enhance the LMD-ed part’s quality is integrating multi-physics mathematical simulations in the processing. LMD is defined by the countless physical processes such as thermal heat transfer, particle melting followed by solidification, evaporative heat transfer, particles interaction, beam energy density and material interaction, thermo-capillarity phenomena and recoil pressures. Using a verified mathematical method, one can investigate the effect of overall flow and heat transfer economically. Two different computational methods have been applied in the literature: one set of models is specifically designed for layers’ deposition. In contrast, the other has been applied to simulate thermo-mechanical phenomena within the layers. The latter has been employed to analyze stress concentration, strain, and the ultimate deflection of a part corresponding to its nomenclature. Farahmand et al. [28] and Bucă et al. [29] observed the influence of laser scanning speeds on the intensity of the final stress concentration by developing a thermo-mechanical finite element (FE) model. The model was able to estimate results close to the experimental ones with a few deviations due to the exclusion of fluid flow in the developed model. Hao et al. [30] designed a simulation model to examine the thermal fields using inverse method calibration, using conduction-based modelling. Nonetheless, Kamara et al. [31] found that pure conduction models are not trustworthy without a complex anisotropic enhanced conductivity. As a result, many scientists have included fluid dynamics calculations in their simulations [32,33,34]. Gan et al. [35,36] developed a Computational Fluid Dynamics (CFD) framework to model the clad morphologies for single and multi-layers for the direct energy deposition process. The model forecasted the resultant microstructure (single and multi-layers) in various steel alloys. The Marangoni force and pressures owing to interfacial tension were also considered in their study. For Ni-based super-alloy IN713-LC, Mohsin Raza et al. [37] determined that with optimum laser power and scanning speed, one can get the highest relative density parts with almost no cracks. Dezfoli et al. [38] developed a simulation framework consisting of a 3D finite element model and a cellular automaton model for predicting the epitaxial grain growth in the single-track LPBF processing of IN718.

In a recent study, Zhao et al. [39] employed a coupling stage configuration and fluid volume approach to simulate the free surface using the Finite Volume Method (FVM). Kumar et al. [40] came up with hydrodynamic and thermal modelling of the mechanism. They conducted a dimensionless investigation on the influence of the Marangoni force on the melt pool morphology. As per Farahmand et al. [28], a major impact on the melt pool is indeed the Marangoni force. Almost all previous papers that dealt with powder motion overlooked the metal powder and assumed the Gaussian distribution of powder mass sufficient to model the particles [35,36,39,40]. Numerous studies on molten metal have only explored the movement of the particles, ignoring the movement and temperature distribution within the melt pool [41,42,43,44]. A research effort was carried out by Sojka et al. [44], who employed CFD-Discrete Phase Method (DPM) to simulate the laser cladding. Bedenko et al. [45] and Kovalev et al. [46] presented a model that analyzed the powder trajectories and flows simultaneously. The impact of the particles over the molten pool was also selectively considered in their research since fluid dynamics was not considered. Instead, a surface development scenario was used to determine the deposited shapes [46]. Both Ishimito et al. [47] and Nie et al. [48] utilized a similar model to the fluid dynamics. They also explained the velocity of the metal powder and melt pool movements during the layer deposition.

In this research, mathematical and computational fluid dynamics (CFD) models for the single-layer deposition of Ti6Al4V alloy were proposed. A simplified mathematical model has been presented for analytical modelling to calculate the single layer dimensions deposited on the substrate using the primary operating conditions. In the case of CFD modelling, the volume of fluid (VOF) and discrete element modelling have been incorporated. The results attained by both models were compared with the single-layer depositions of Ti6Al4V attained via LMD experiments. Furthermore, in the LMD process, a mechanism for detecting the flow behavior within the whole melt pool has been developed. The melt pool’s overall flow behavior was also revealed. The driving elements of liquid flow and fundamental processes have been explored throughout the melt pool. Besides, the mass flow rate explains the equivalent flow caused by the Marangoni effect.

## 2. Modelling

This section has been divided into two parts: (a) analytical modelling and (b) computational fluid dynamics (CFD) modelling.

### 2.1. Analytical Modelling

When determining the geometry of the deposited layer, the following assumptions were taken into consideration:

1. The gravitational effect during the powder particle flow is neglected. This assumption is reasonable as the time of flight of powder particles across the laser beam interaction zone is very short, equivalent to 25% of standoff distance [49].
2. Powder particle impact forces on the geometrical properties of the clad are ignored. The capillary action was disregarded during the 3D printing in the analytical modeling. Powder flow is considered stable with constant thermo-physical properties.
3. For analytical modelling, the boundary conditions for heat losses by convection and radiations have been ignored, while laser energy losses have been considered via laser beam absorption coefficient.

Here, the Beer-Lambert law has been applied to express the powder deposition in the case of a translating laser beam as:

(1) $$\frac{m_{o}C^{*}\Delta T}{\pi t_{o}r_{p}^{2}}\left[\frac{J}{sm^{2}}\right]=I_{o}e^{-\alpha ℵh}\left[\frac{W}{m^{2}}\right]$$

Here, $m_{o}$ is the mass printed on the substrate as a result of melting, C* is modified heat capacity of the powder material, ΔT the thermal gradient from room temperature to an elevated temperature, *t_o_* is the laser-material interaction time, *r_p_* is the focused laser beam spot size, $I_{o}$ the laser beam intensity interacted with the material, α is the laser beam absorption fraction, *h* the height along the universal *z*-axis in the LMD process and ℵ is the gloom of the particles being added with the beam. The term ℵ can be altered spatially by overlapping powder and beam interaction, thus causing the laser’s beam to be attenuated and calculated as [50]:

(2) $$ℵ=\frac{M_{p}}{\pi r_{pn}^{2}v_{p}},$$

where *M_p_* can be described as the powder flow rate, *r_pn_* is the nozzle radius of powder outlet, and *v_p_* is speed of the powder debits. Using mathematical formalism, C* is expressed:

(3) $$C^{*}=\frac{L_{f}}{T\left(y,t\right)-T_{o}}+C.$$

Here, *L_f_* is the fusion enthalpy of debits, *C* is the powder material heat capacity, *T_o_* is expressed as ambient temperature, while *T*(*y*,*t*) is the one-dimensional transient temperature. The intensity of a Gaussian laser beam having laser power (*P*) is written as [51]:

(4) $$I_{o}=\frac{2\forall_{1}P}{\pi r_{p}^{2}}.$$

In the LMD printing, laser energy density is utilized and dispersed through the debits as they pass by the laser intensity, thus causing laser energy diminution ( $\forall_{1}$ ) at substrate. In the previous studies of the authors [52], an analytical formula was deduced to estimate $\forall_{1}$ , as:

(5) $$\forall_{1}=\frac{3M_{p}\forall_{2}0.25\left(SOD\right)}{4\pi r_{pp}^{2}\rho_{p}v_{p}r_{p}^{2}}.$$

In Equation (5), $\forall_{2}$ is powder utilization efficiency during the deposition process, and *r_pp_* and $\rho_{p}$ are the powder particles’ mean radius and density, respectively. In LMD printing, the final deposited powder layer mass ( $m_{o}$ ) is affected by the laser beam speed (*V_s_*), laser-material interaction (*t_o_*) and final printed layer (*L*), as:

(6) $$m_{o}=m\frac{V_{s}t_{o}}{L}.$$

Here, *m* is the powder mass passing under the laser beam. The thermal gradient ( ΔT ) is defined as:

(7) $$\Delta T=T\left(y,t\right)-T_{o},$$

After substituting the Equations (2)–(7), rearranging, and applying natural log, one can get the following expression:

(8) $$h=\left|-\frac{\pi r_{pn}^{2}v_{p}}{\alpha M_{p}}ln\left(\frac{mV_{s}t_{o}\left(\frac{L_{f}}{T\left(y,t\right)-T_{o}}+C\right)\left(T\left(y,t\right)-T_{o}\right)}{2\left(\frac{3M_{p}\forall_{2}0.25\left(SOD\right)}{4\pi r_{pp}^{2}\rho_{p}v_{p}r_{p}^{2}}\right)\forall_{1}PL}\right)\right|.$$

The *h* value can be estimated if *T*(*y*,*t*) is known. Iacobescu [53] presented an analytical solution to determine *T*(*y*,*t*) in the laser welding; however, this solution has been modified for the LMD process. Two new factors have been introduced in the *T*(*y*,*t*) solution: (a) $\forall_{1}$ and (b) ℵ .

(9) $$T\left(y,t\right)=T_{o}+\frac{\sqrt{3}}{2\rho_{s}C_{s}\sqrt{\pi }}\frac{Q\forall_{1}\sqrt{ℵ}}{\sqrt{12\alpha t+r_{p}^{2}}}exp\left[-\frac{y^{2}}{4\alpha t}+\frac{y^{2}r_{p}^{2}}{4\alpha t\left\{12\alpha t+r_{p}^{2}\right\}}\right]erf\left[\frac{12\alpha t-yr_{p}+r_{p}^{2}}{2\sqrt{ℵ\alpha t\left\{12\alpha t+r_{p}^{2}\right\}}}\right]+exp\left[-\frac{y^{2}}{4\alpha t}+\frac{y^{2}r_{p}^{2}}{4\alpha t\left\{12\alpha t+r_{p}^{2}\right\}}\right]erf\left[\frac{12\alpha t+yr_{p}+r_{p}^{2}}{2\sqrt{ℵ\alpha t\left(12\alpha t+r_{p}^{2}\right)}}\right]$$

In Equation (9), $\rho_{s}$ is the substrate’s density, $C_{s}$ is the heat capacity of substrate, *Q* is the thermal energy per unit area, α is the material’s thermal diffusivity, *t* is the total laser-substrate interaction time, *t_p_* is the waiting time between two depositions, *erf* is an error function that occurred when integrating a normalized distribution [54] and *n* is the number of layers deposited on a substrate. Considering an elliptical shape of the deposited layer, if *h* is known, the width (*w*) and depth (*d*) of a single layer are determined as [52]:

(10) $$w=\frac{4\forall_{2}M_{p}}{\pi hV_{s}\rho_{p}}.$$

(11) $$d=\frac{\left[\forall_{2}\left(1-\forall_{1}\right)\left(PL-V_{s}\right)\right]-\left[\forall_{2}M_{p}LC^{*}\right]}{\left[\frac{\pi }{6}\rho_{s}wLC_{s}^{*}\right]}.$$

In Equation (11), *L* is the length of the deposited layer and $C_{s}^{*}$ is the modified specific heat of substrate, defined as:

(12) $$C_{s}^{*}=\frac{L_{fs}}{T_{ms}-T_{o}}+C_{s}.$$

### 2.2. Numerical Modelling: CFD

**Figure 1:**
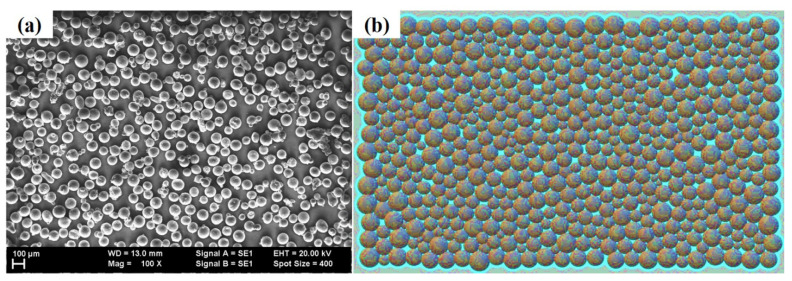
Ti6Al4V particulates with 50–130 µm particle size distribution (**a**) SEM and (**b**) computational results.

**Figure 2:**
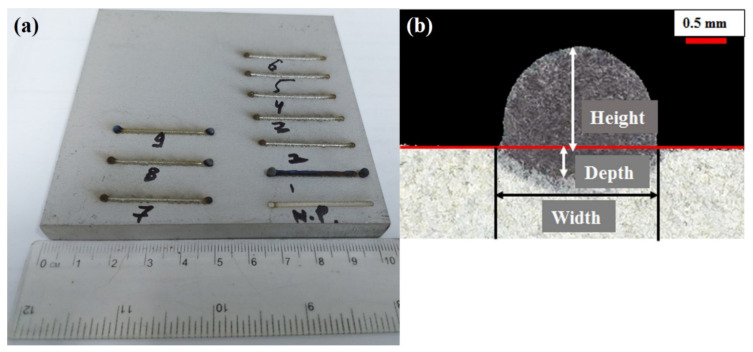
(**a**) Nine Ti6Al4V single tracks deposited on Ti6Al4V substrate and (**b**) a typical cross-section of an LMD deposited layer.

Following steps were implemented for CFD modeling:

- Initially, many particles fall concurrently with the translating laser scanning head. Here, the debits are heated and melted, resulting in a layer formation. The elastic real contact force for powder particles is measured using an interactive approach based on the Hertz–Mindlin formalism [55]. Simultaneously, the damping factor accounts for mechanical energy dissipation [56,57,58].
- Elastic materials have natural contact and damping forces that overlap in the perpendicular plane between interacting particles. The mass and Young’s modulus of the given material are considered equivalent. No micro-slip technique is used to handle the elastic contact force [55].
- The FS-DEM module from Flow Science, USA was utilized to conduct the deposition of Ti6Al4V powder particles on Ti6Al4V substrate. Discrete micro-particles were used to deposit the powder layer. Figure 1a,b illustrate an evaluation of powder debits distribution obtained by the scanning electron microscopy (SEM, Carl Zeiss, Oberkochen, Germany) and software (Flow 3D by Flow Science, Santa Fe, NM, USA), correspondingly. Ti6Al4V particulates were between 50–130 µm, as shown by SEM in Figure 2a, and the computed particle size distribution obtained from the numerical model is shown using Figure 2b.

- The rapid melting leading to solidification of a specific material in the LMD process influences the thermo-physical properties of a given material. Temperature-dependent thermo-physical characteristics of Ti6Al4V with phase shifts were chosen for the CFD model.
- The boundary conditions for heat losses such as convection and radiation were introduced via the Energy balance equation for CFD simulations.

The CFD work environment was built and executed using the commercial FLOW-3D CFD module and specific sub-processes. During LMD printing, the melting flow is incompressible Newtonian, and the variation in mass owing to vaporization is omitted. Speed and temperature profile fields were computed via solving balance-of-mass, linear momentum, and energy balance partial differential equations within the fusion zone [59,60]. Additionally, an aggregated body approximation-based energy balance formula was used to calculate the heat of the metal powder.

#### 2.2.1. Motion Equations

To find the velocity and pressure fields, a composite system solving the mass balance and momentum equations were employed [61]:

(13) $$\vec{\nabla }\cdot \vec{V}=0.$$

(14) $$\begin{array}{cc} \rho \left[\frac{\partial }{\partial t}\left(\vec{V}\right)+\vec{V}\cdot \vec{\nabla }\left(\vec{V}\right)\right]= & -\vec{\nabla }p+\vec{\nabla }\cdot \left[\mu \left(\vec{\nabla }\vec{V}+\vec{\nabla }{\vec{V}}^{T}\right)-\frac{2}{3}\delta_{ij}\vec{\nabla }\cdot \vec{V}\right]\\ & -\frac{C_{1}{\left(1-f_{liquid}\right)}^{2}}{C_{2}+f_{liquid}^{3}}\vec{V}-\rho \vec{g}\beta \left(T-T_{liquid}\right). \end{array}$$

In Equation (14), the second term is linked to viscous shear stresses, while C_1_ and C_2_ are the constants related to drag forces found during solidification [62]. The final term in Equation (14) is tied to buoyancy. Free surfaces of the fluid are tracked by using the VOF approach [63]. Equation (15) uses the fluid fraction *F* to describe how much fluid is inside a computational cell:

(15) $$\rho \frac{\partial }{\partial t}\left(F\right)+\vec{V}\cdot \vec{\nabla }\left(F\right)=0.$$

A zero *F* value signifies the cell is without fluid, and a unity *F* value suggests the cell is filled with the fluid. Additionally, a moderate *F* reveals the fluid interface’s location. Free surfaces are influenced by three temperature-related surface tractions when operating at high temperatures:

(16) $$\tau_{capillary}=\left(\sigma_{ref}-\gamma \left[T-T_{ref}\right]\right)k.$$

(17) $$\tau_{Marangoni}=\gamma \left[\vec{\nabla }T-\left(\vec{\nabla }T\cdot \vec{n}\right)\vec{n}\right],$$

(18) $$\tau_{recoil}=0.54P_{0}\exp\left[\frac{\Delta H_{lv}}{R_{v}\cdot T_{boiling}}.\left[1-\frac{T_{boiling}}{T}\right]\right]\vec{n}.$$

where the surface tension reference (*σ_ref_*) shows the sensitivity of surface tension with respect to temperature. Equations (16)–(18) have been taken from Refs. [58,64,65]. The curvature and the normal vector can be obtained via the subscript *n* in the exposed liquid:

(19) $$\vec{n}=\frac{-\vec{\nabla }F}{\left|\vec{\nabla }F\right|}.$$

Surface tension and rebound pressure operate on the liquid metal perpendicularly to each other. In contrast, the Marangoni effect works on the opposite side of the free surface, as shown in Equation (17).

#### 2.2.2. Energy Balance Equation

Solving the energy balance equation is necessary to identify the heat within the weld zone, and its fusion expressed as:

(20) $$\rho \left[\frac{\partial }{\partial t}\left(h\right)+\vec{V}\cdot \vec{\nabla }\left(h\right)\right]=\vec{\nabla }\cdot \left[k\vec{\nabla }T\right],$$

The fluid velocity vector *V* is influenced by the metal density (*ρ*) and thermal conductivity (*k*) as shown in Equation (20). The metallic material’s enthalpy (*h*) is specified as a function of temperature:

(21) $$h\left(T\right)=h_{ref}+\int_{T_{ref}}^{T}C_{p}\cdot dT+f_{liq}\cdot \Delta H_{sl},$$

where Δ*H_sl_* is the latent heat of fusion, *h_ref_* is the enthalpy, and *C_p_* is the specific heat capacity. In this study, the relationship between temperature and melting/solidification is taken as linear [66,67], expressed as:

(22) $$\begin{array}{c} 1\\ \left(T-T_{solid}\right)/\left(T_{liquid}-T_{solid}\right)\;;\;\\ 0 \end{array}\begin{array}{c} ;T\geq T_{liquid}\\ T_{solid}<T<T_{liquid}\\ ;T\leq T_{soluid} \end{array},$$

Equation (22) is denoted by the subscripts solid and liquid, which signifies the solid and liquid phases, respectively. In the case of phase evolution, *k*, *C_p_* and *ρ* are obtained using a rule of mixture [68]:

(23) $$k=f_{sol}k_{sol}+f_{liq}k_{liq}.$$

(24) $$C_{p}=\frac{f_{solid}\rho_{solid}C_{p{,}_{solid}}+f_{liquid}\rho_{liquid}C_{p{,}_{liquid}}}{f_{solid}\rho_{solid}+f_{liquid}\rho_{liquid}}.$$

#### 2.2.3. Powder Debits

It is necessary to calculate another set of the energy balance equations based on the lumped body approximation to determine the temperature of the powder particle [69]:

(25) $$\rho =f_{solid}\cdot \rho_{solid}+f_{liquid}\cdot \rho_{liquid}.$$

(26) $$m_{p}C_{p}\frac{dT_{p}}{dt}=h_{p}A_{p}\left[T_{sr}-T_{p}\right]+\varepsilon_{p}\eta A_{p}\left[T_{sr}^{4}-T_{p}^{4}\right]+A_{p}^{\prime }q_{laser}^{\prime \prime }.$$

where *m_p_* is the powder mass, *A_p_* is the surface area of a single powder particle, and *h_p_* and *ε_p_* are the convection heat transfer constant and emissivity of the debit material, respectively. Furthermore, *T_sr_* is the ambient temperature (= 25 °C), *η* is Stefan-Boltzmann constant and $q_{laser}^{\prime \prime }$ is the laser energy intensity responsible for melting. Here, the mass flow rate ( $\dot{m}$ ) is calculated as:

(27) $$\dot{m}=\int \;\rho \cdot \vec{v}d\vec{A}.$$

Here, $\vec{v}$ is velocity and  ρ is density. Simulation values were supposed for width and height to reflect a close change with experimental results and do not contain real measurements in the simulations. Simulation values for mass flow rate are assumed based on the results obtained in single CFD visualization.

## 3. Materials and Methods

For validations, LMD equipment from KR30HA, Germany, having a Yb: YAG laser source, was utilized to deposit single tracks of Ti6Al4V powder particles on Ti6Al4V substrate samples. In one of the recent studies by Chioibasu et al. [70], Ti6Al4V implants were manufactured by the LMD process. To print these prototypes, Ti6Al4V substrate was utilized. The metallographic investigations and X-ray diffraction data exposed an unusual biphasic α+β structure. The in-vitro tests performed on the manufactured Ti6Al4V samples in osteoblast-like cell cultures up to 7 days showed that the material deposited by laser melting is cytocompatible. For this study, single tracks of Ti6Al4V were deposited on Ti6Al4V substrate. The laser spot size was 800 µm with super-Gaussian energy distribution within the spot. Each of the three parameters—scanning velocity, debit flow rate and power—was adjusted to classify the final layer’s thickness and width. The baseplate (substrate) geometry were: length = 100 mm, width = 100 mm and thickness = 10 mm. Table 1 displays the operating conditions for nine single-track experiments of Ti6Al4V depositions.

Table 2 collects the thermo-physical properties of Ti6Al4V material.

The deposited layers’ height and width were measured and documented to compare the experimental results with simulation models. Figure 2a shows the nine single tracks of Ti6Al4V deposited on Ti6Al4V substrate, while a cross-section of a typically deposited layer is presented in Figure 2b. In Figure 2a, N.P. shows the single scan carried out without coaxial powder addition to identify a clear difference on single layer dimensions in the case of with and without powder material. To determine the deposited layer’s height and width, all the samples were prepared according to the cross-section provided in Figure 2b, and the dimensions were recorded using optical microscopy.

## 4. Results and Discussions

**Figure 3:**
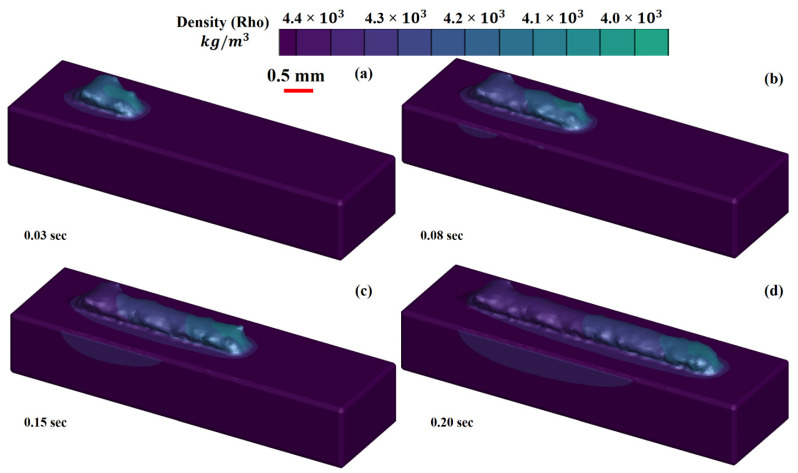
Three-dimensional view of the deposited layer with density evolution in the LMD deposition of Ti6Al4V at: (**a**) 0.03 s, (**b**) 0.08 s, (**c**) 0.15 s and (**d**) 0.20 s.

Figure 3a–d show the melt pool states generated at time intervals of (a) 0.03 s, (b) 0.08 s, (c) 0.15 s, and (d) 0.28 s, as well as the cooling of the deposited material from melting to ambient temperature (d). It can be demonstrated that, as the temperature rises, the density of the material rapidly decreases due to the heat capacity and latent heat, thus increasing the fluid volume. It is important to note that the volume increases dramatically due to a drop in density, resulting in surface tension declination. It is the differential in surface tension that influences the melt pool dimensions. When the surface tension between two ends of a liquid is developed, a strong pull-force is generated from the high to the low surface tension end, known as the “Marangoni effect.” Due to surface tension differences, a large pulling force is generated from one end to another. Figures show that when the layer is printed on the substrate, heat begins to dissipate from the deposited layer to the substrate, causing a change in the density of the substrate.

**Figure 4:**
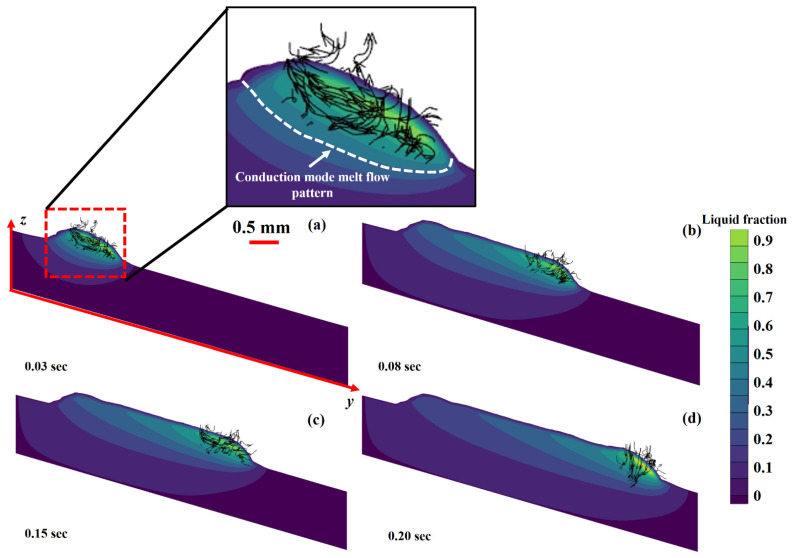
Section view of the LMD deposited layer from the top view in the case of Ti6Al4V material at various intervals: (**a**) 0.03 s, (**b**) 0.08 s, (**c**) 0.15 s and (**d**) 0.20 s.

Two types of melt flow patterns have been observed during laser-material interaction: (a) conduction region (CR) and (b) depression region (DR) [72,73]. For CR, a melt pool is formed when a substance is heated to its melting point using laser energy that exceeds the rate at which heat is dissipated. In the DR, laser energy from the heating source concentrates to such a high degree that the material’s melting and boiling points are exceeded. Because of the material’s vaporization, the melt pool experiences a rebound pressure, resulting in the DR. The DR is also categorized as “keyhole.” This research has only found evidence of CR formation. The LMD deposition technique involves the addition of powder particles simultaneously. The powder particles use a considerable fraction of the laser beam energy to change their phase from solid to liquid. In turn, it reduces the net amount of laser energy arriving at the substrate, resulting in only conduction-mode melt flow [52,74]. Figure 4a–d exhibits the section view of the LMD-ed layer, and CR melt-pool can be observed. In the Ti6Al4V deposition on a Ti6Al4V substrate, the simulations were carried out using a power equal to 900 W, scanning velocity equal to 0.015 m/s, and a debit feeding rate equal to 5.0 g/min. In laser additive manufacturing, five driving forces, including Marangoni force, recoil pressure of vaporization, a shear force due to high-speed vapor cloud, hydraulic pressure, buoyancy force [74]. Marangoni force” flows the material from an elevated to a low thermal domain [75,76,77]. The recoil pressure of “vaporization” implements a compression, internally, transverse to the face experiencing the evaporation [78,79]. Shear force can be produced by a “high-speed vapor cloud” due to resistance at the gas-liquid interface [80]. ”Hydraulic pressure” can transmit energy in two ways: hydrostatic and hydrodynamic pressures [81,82,83]. The “buoyancy force” forces the molten substance to follow the density gradient [81,84,85]. Besides, convection is the primary mode of heat transport in the molten pool controlled by the primary operating conditions [86,87,88]. The above-defined forces are responsible for defining the melt flow patterns.

**Figure 5:**
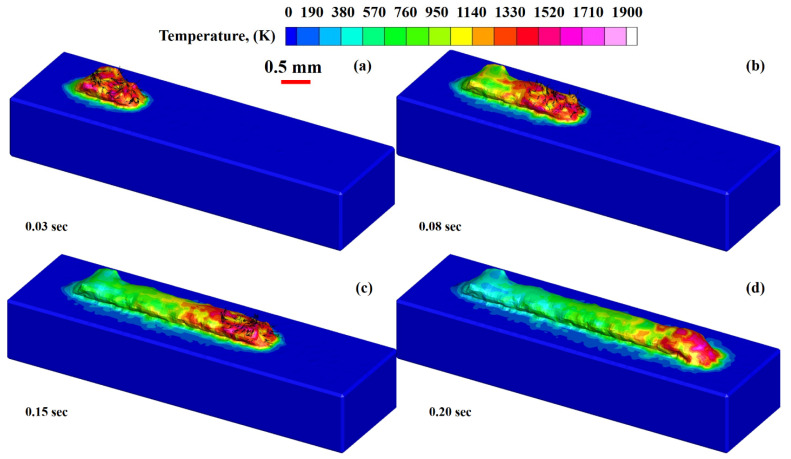
Temperature distribution of the printed layer at various intervals: (**a**) 0.03 s, (**b**) 0.08 s, (**c**) 0.15 s and (**d**) 0.20 s.

Figure 5a–d compiles the evolution of thermal distribution during the printing of Ti6Al4V layer on Ti6Al4V substrate. During LMD additive manufacturing, a 1900 K temperature was achieved. During deposition, the material temperature dropped from melting to ambient temperature due to the printed layer’s heat losses, resulting in layer deposition. It is essential to emphasize that the extreme layer area is in contact with ambient air, which is responsible for the heat elimination from the deposited layer’s top. 

**Figure 6:**
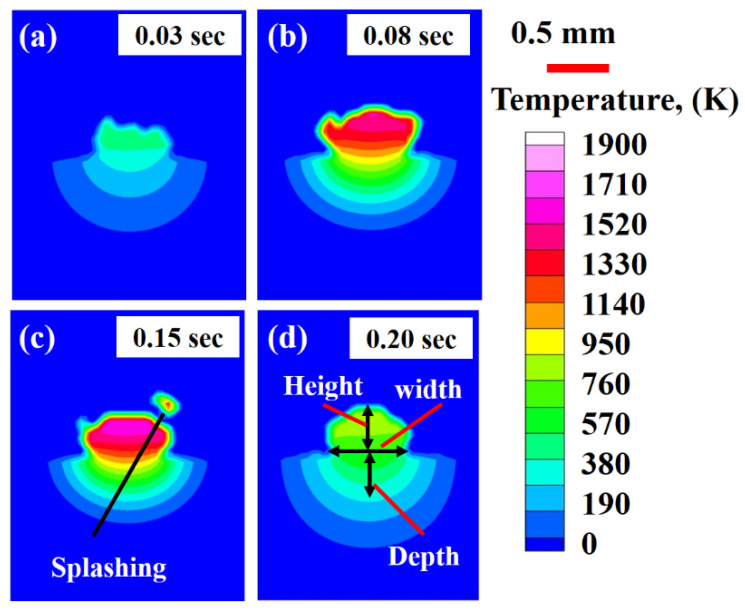
Thermal distribution at laser beam location in the Ti6Al4V cross section deposited by LMD at: (**a**) 0.03 s, (**b**) 0.08 s, (**c**) 0.15 s and (**d**) 0.20 s.

Figure 6a–d display the evolution of thermal distribution inside the deposited layer cross-section at 0.03 s, 0.08 s, 0.15 s and 0.20 s periods. The results have been presented at laser beam location during layer printing at different time domains. The deposited layer’s height, width, and depth can be identified during the deposition. From Figure 6c, it can be observed that a droplet of molten material was eliminated from the previously deposited layer. In LMD, the debits involve two kinds of heatings (a) in-flight and (b) inside the generated molted pool [74]. During deposition, in-flight heated particles, a few partially melted, try to enter into the molten pool generated via a laser beam within the base plate. Upon ejecting from the powder nozzle output, the debits are focused on the molten pool, thus adopting a Gaussian shape. However, they experience collisions that increase the powder particles’ chances to fall onto the previously deposited layer. This phenomenon causes splashing within the melt material during the LMD deposition process.

**Figure 7:**
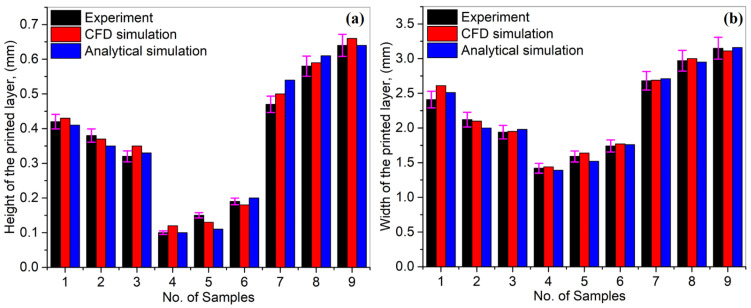
A comparison for **(a)** height and **(b)** width in the case of Ti6Al4V experiments and simulations.

Figure 7a,b show a comparison among experiments, CFD simulation and analytical simulation results in the case of layer height and width of Ti6Al4V LMD-ed layers. A close link between experiments and CFD modelling was determined with a 1–3% mean absolute deviation. However, the analytical model showed results with 9–12% variation. A higher deviation value is due to the negligence of surface tension and the 40% powder utilization efficiency applied during analytical simulations. A much-reduced error value demonstrates the dependability of the CFD and analytical models for the LMD process estimation.

**Figure 8:**
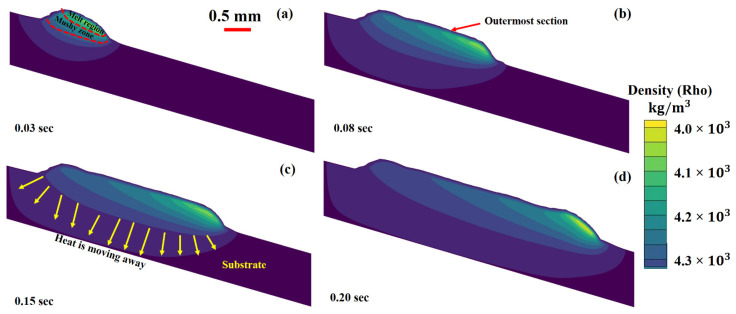
Cross section for the liquid to solid conversion in Ti6Al4V layer deposition at: (**a**) 0.03 s, (**b**) 0.08 s, (**c**) 0.15 s and (**d**) 0.20 s.

The cross-sections for liquid and solid transformations at various periods 0.03 s, 0.08 s, 0.15 s and 0.20 s are shown in Figure 8a–d. Conduction, convection and radiation are responsible for heat elimination from the LMD printed layer. The heat inside the metallic material causes conduction. Three regions have been recognized in Figure 8: (a) molten regime, (b) mushy (solid + liquid mixture) area and (c) solidified regime [74]. The molten and mushy areas are critical for elaborating the microstructure formation and mechanical-physical qualities. 

**Figure 9:**
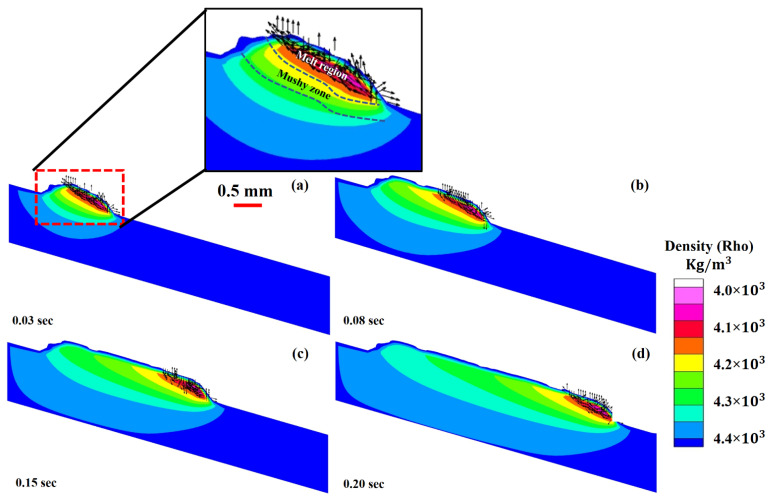
Deposited layer cross section having velocity vectors with liquid-solid transformation at: (**a**) 0.03 s, (**b**) 0.08 s, (**c**) 0.12 s, and (**d**) 0.15 s.

Ti6Al4V deposited layer’s velocity vectors were studied at various time intervals as shown in Figure 9a–d. Here, the color code is used to distinguish the melt pool densities. As the laser beam starts irradiating the base plate with the simultaneously powder debits feeding, the melt material is forced to move backwards due to recoil pressure and Marangoni effect. A reaction force was produced at the laser-surface periphery due to velocity vectors. When the beam moves away from the heated region, the molten region, which is in direct contact with the air, begins to cool, resulting in a significant surge in surface tension. It can be analyzed here that the velocity trajectories near the surface are dragging the liquid higher.

## 5. Conclusions

In this paper, analytical and CFD models have been developed for the single-layer deposition of Ti6Al4V alloy. For CFD modelling, the volume of fluid (VOF) and discrete element modelling approaches have been utilized, while simplified mathematical equations have been deduced in the case of an analytical model. Furthermore, a methodology has been designed to identify the generated molten pool’s flow pattern and its dynamics. In addition, the factors that drive liquid flow and fundamental processes have been identified. Experiments have been performed for single-layer deposition of Ti6Al4V using LMD equipment. Experiments and simulations have been found to have a close connection with a deviation of 1–3% for CFD modelling and 9–12% for analytical modelling. Based on the current investigation, the following conclusions have been drawn:

- In laser additive manufacturing, there are two melt flow patterns: (a) conduction region (CR) and (b) depression region (DR). However, only CR melt flow has been simulated in the LMD deposition process.
- The simulation results showed that the molten material droplet was eliminated from the deposited layer. During printing, a few partially melted in-flight heated particles try to enter into the molten pool, thus, causing splashing within the melt material.
- The density of a given substance rapidly lowers as the temperature rises due to the material’s heat capacity and latent heat, thus elevating the fluid volume. The surface tension (ST) differential is critical in determining the melt flow pattern. A variation in ST causes the development of a “Marangoni” force.
- It was simulated that heat escapes through conduction, convection and radiation when the layer is deposited. The melt regime, mushy area and solidified regime were identified in LMD printing. Due to recoil pressure and the Marangoni effect, melt flow is compelled to flow backward when the laser energy commences the substrate irradiation. As the beam moves forward, melt flow is dragged along by the increased capillary action.
- By simulations, it has been found that analytical models are more efficient than CFD ones. However, they give results with a higher deviation (9–12%) than the experimental values and cannot show an in-depth melt flow field. On the other hand, CFD models can yield an in-detail melt flow field with accuracy up to 1–3% compared to the experimental analyses at the cost of much higher computational time.

## Figures and Tables

**Table 1:** Ti6Al4V single layer depositions using LMD set-up.

Specimen Number	Power (W)	Scanning Velocity (m/s)	Debit Flow Rate (g/min)	Helium/Argon Gases (bar)
01	700	0.005	3.0	3.0/7.0
02	700	0.015	3.0	
03	700	0.025	3.0	
04	500	0.005	2.0	
05	500	0.005	3.0	
06	500	0.005	5.0	
07	500	0.015	5.0	
08	700	0.015	5.0	
09	900	0.015	5.0	

**Table 2:** Thermo-physical properties of Ti6Al4V (data from Ref. [71]).

Sr. No.	Property Name	Value (Unit)
1	Density	4.4 × 10^3^ kg/m^3^
2	Poisson’s ratio	0.31
3	Young’s Modulus	110 GPa
4	Latent heat of fusion	360 kJ/kg
5	Melting temperature	1878 K
6	Specific heat	553 J/kgK
7	Thermal conductivity	7.1 W/mK
8	Thermal expansion	8.7 × 10^−6^ /K

## Data Availability

Not applicable.
