# Correction: Mahmood et al. Laser Melting Deposition Additive Manufacturing of Ti6Al4V Biomedical Alloy: Mesoscopic In-Situ Flow Field Mapping via Computational Fluid Dynamics and Analytical Modelling with Empirical Testing. *Materials* 2021, *14,* 7749

**DOI:** 10.3390/ma19050968

**Published:** 2026-03-03

**Authors:** Muhammad Arif Mahmood, Asif Ur Rehman, Fatih Pitir, Metin Uymaz Salamci, Ion N. Mihailescu

**Affiliations:** 1 National Institute for Laser, Plasma and Radiation Physics (INFLPR), Magurele, 077125 Ilfov, Romania; ion.mihailescu@inflpr.ro; 2 ERMAKSAN, Bursa 16065, Turkey; 3 Department of Mechanical Engineering, Gazi University, Ankara 06570, Turkey; 4 Additive Manufacturing Technologies Research and Application Center-EKTAM, Gazi University, Ankara 06560, Turkey; 5 Manufacturing Technologies Center of Excellence-URTEMM A.S., Ankara 06980, Turkey

**Missing Citation**

In the original publication [1], the citations “Bayat, M.; Nadimpalli, V.K.; Biondani, F.G.; Jafarzadeh, S.; Thorborg, J.; Tiedje, N.S.; Bissacco, G.; Pedersen, D.B.; Hattel, J.H. On the Role of the Powder Stream on the Heat and Fluid Flow Conditions during Directed Energy Deposition of Maraging Steel—Multiphysics Modeling and Experimental Validation. *Addit. Manuf.* **2021**, *43*, 102021. https://doi.org/10.1016/J.ADDMA.2021.102021” and “Bayat, M.; Thanki, A.; Mohanty, S.; Witvrouw, A.; Yang, S.; Thorborg, J.; Tiedje, N.S.; Hattel, J.H. Keyhole-Induced Porosities in Laser-Based Powder Bed Fusion (L-PBF) of Ti6Al4V: High-Fidelity Modelling and Experimental Validation. *Addit. Manuf.* **2019**, *30*, 100835. https://doi.org/10.1016/J.ADDMA.2019.100835” were previously overlooked and are newly added in the revised manuscript. These two additional references have now been cited in Section 2.2 (Numerical Modelling: CFD, Paragraph 2), which should read as follows:

“The CFD work environment was built and executed using the commercial FLOW-3D CFD module and specific sub-processes. During LMD printing, the melting flow is incompressible Newtonian, and the variation in mass owing to vaporization is omitted. Speed and temperature profile fields were computed via solving balance-of-mass, linear momentum, and energy balance partial differential equations within the fusion zone [59,60]. Additionally, an aggregated body approximation-based energy balance formula was used to calculate the heat of the metal powder.”

With the addition of the two new references and their citations, the order of some references has been adjusted accordingly.

**Text Correction**

There were errors in the original publication [1]. The citations of the following references have been changed to the name of the first author.

In Section 1 (Introduction, paragraph 1), “Paul et al. [18,19,20,21]” has been changed to “Ya et al. [18], Froend et al. [19], Jhavar et al. [20] and Bailey et al. [21]”, and “Liu et al. [22]” has been changed to “Zhu et al. [22]”.

In Section 1 (Introduction, paragraph 2), “Kovacevic et al. [28] and Buca et al. [29]” has been changed to “Farahmand et al. [28] and Bucă et al. [29]”, “Marimuthu et al. [31]” has been changed to “Kamara et al. [31]”, and “Raza et al. [37]” has been changed to “Mohsin Raza et al. [37]”.

In Section 1( Introduction, paragraph 3), “Rong et al. [39]” has been changed to ‘’Zhao et al. [39]”, “Roy et al. [40]” has been changed to “Kumar et al. [40]”, “Kleijin et al. [28]” has been changed to “Farahmand et al. [28]”, “Kovalev et al. [45,46]” has been changed to “Bedenko et al. [45] and Kovalev et al. [46]”, and “Li et al. [48]” has been changed to “Nie et al. [48]”.

The authors state that the scientific conclusions are unaffected. This correction was approved by the Academic Editor. The original publication has also been updated.
